# Cerebral autosomal dominant arteriopathy with subcortical infarcts and leukoencephalopathy (CADASIL) with multiple different onset forms of frequent recurrent attacks: A case report and literature review

**DOI:** 10.1097/MD.0000000000037563

**Published:** 2024-03-15

**Authors:** Siting Wu, Ning Zhao, Tingting Sun, Fang Cui, Xianli Sun, Jiacai Lin

**Affiliations:** a Department of Neurology, Hainan Hospital of Chinese PLA General Hospital, Sanya, China; b Department of Orthopaedics, Hainan Hospital of Chinese PLA General Hospital, Sanya, China; c Department of Health Medicine, Hainan Hospital of Chinese PLA General Hospital, Sanya, China.

**Keywords:** case report, cerebral autosomal dominant arteriopathy with subcortical infarcts and leukoencephalopathy, cerebral white matter lesions, NOTCH3

## Abstract

**Introduction:**

Cerebral autosomal dominant arteriopathy with subcortical infarcts and leukoencephalopathy (CADASIL) is one kind of monogenic hereditary small-vessel disease in the brain caused by mutations in the NOTCH3 gene. However, it is rare for CADASIL to recur with different clinical manifestations in 1 patient, and some atypical clinical manifestations can easily lead to misdiagnosis by clinical physicians.

**Case concern:**

A 34-year-old male presented with transient speech disorder accompanied by weakness in the left side of the body for 1 day in June 2020. Magnetic resonance imaging showed acute ischemic infarction in right centrum semiovale, along with multiple abnormal white matter hyperintensities in the brain. Genetic sequencing identified a heterozygous mutation in the NOTCH3 gene. The patient experienced recurrent episodes in 2021 and 2023, with varying clinical symptoms including visual blurring, abnormal limb sensation, and sudden cognitive dysfunction.

**Diagnosis:**

The diagnoses of CADASIL is based on clinical manifestations, imaging results, and genetic reports.

**Intervision and outcomes:**

The patient was received symptomatic treatment including antiplatelet aggregation therapy, lipid regulation, and plaque stabilization, resulting in improved symptoms.

**Outcomes:**

During the course of the disease, after medication treatment and rehabilitation exercise, the patient clinical symptoms have significantly improved. Currently, the patient is closely following up and regularly undergoing relevant examinations.

**Lessons:**

In this rare case, we found that CADASIL can recur multiple times in a patient with different clinical symptoms, which can easily lead to clinical misdiagnosis. Clinicians should consider the possibility of CADASIL in young patients with sudden typical neurological dysfunction.

## 1. Introduction

Cerebral autosomal dominant arteriopathy with subcortical infarcts and leukoencephalopathy (CADASIL) is one kind of monogenic hereditary cerebrovascular disease caused by mutations in the NOTCH3 gene,^[1]^ and is also one of the most common genetic diseases that lead to stroke attacks and cognitive decline. The age of onset for CADASIL is notably broad, with initial symptoms manifesting from early adulthood to the elderly. Although the progression of the disease is relatively slow, its clinical manifestations are diverse, mainly including migraine with typical auras, subcortical ischemic stroke, transient ischemic attacks, psychobehavioral abnormalities, and cognitive impairment.^[2]^ Diagnostic and therapeutic challenges are frequent in clinical settings due to the variability of symptom onset in CADASIL, often resulting in misdiagnosis or inappropriate treatment. Therefore, in order to further enhance the standards of diagnosis and treatment for CADASIL, this paper synthesizes and reviews a case study of a patient with recurrent episodes of CADASIL, treated at the Hainan Hospital of the Chinese People Liberation Army General Hospital, alongside a review of pertinent literature.

## 2. Case presentation

**Figure 1.**
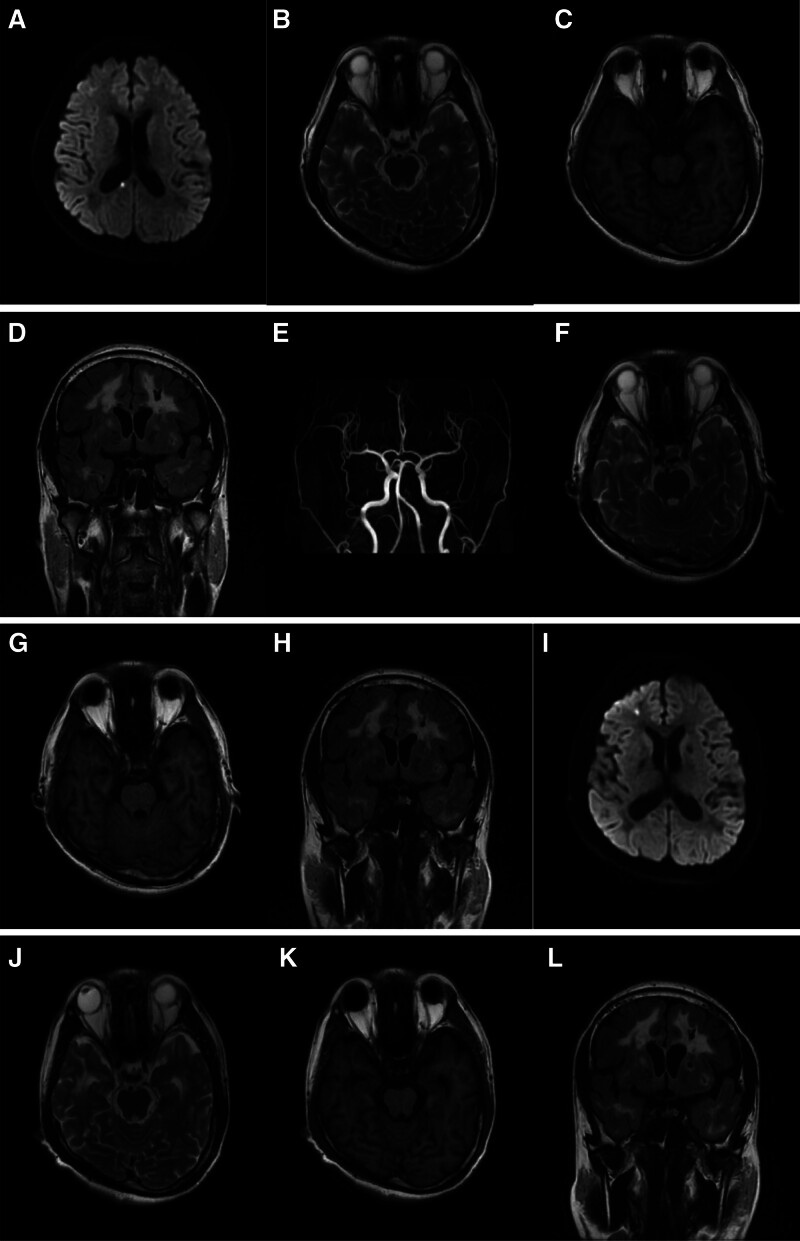
(A) The DWI sequence identified a hyperintense signal in the right corpus callosum, indicative of a potential cerebra acutel ischemic infarction. (B–D) T2-weighted and FLAIR sequences exhibited bilateral, symmetric high-intensity signals around the lateral ventricles and in the deep white matter, along with multiple lacunar strokes involving the external capsule and the temporal pole, with the affected areas presenting slightly hypointense on T1-weighted images. (E) MRA showed no evidence of abnormal changes in the cranial arteries. (F–H) T2-weighted, T1-weighted, and FLAIR sequences displayed an increase in diffuse white matter rarefaction and lacunar infarcts as compared to earlier images (B–D). (I) DWI sequence detected a hyperintense signal in the right frontal lobe, suggestive of an acute cerebral infarction. (J–L) T2-weighted, T1-weighted, and FLAIR sequences revealed a progression in diffuse white matter rarefaction and the number of lacunar infarcts compared to images (F–H). DWI = diffusion weighted imaging, FLAIR = fluid-attenuated inversion recovery, MRA = magnetic resonance angiography.

**Figure 2.**
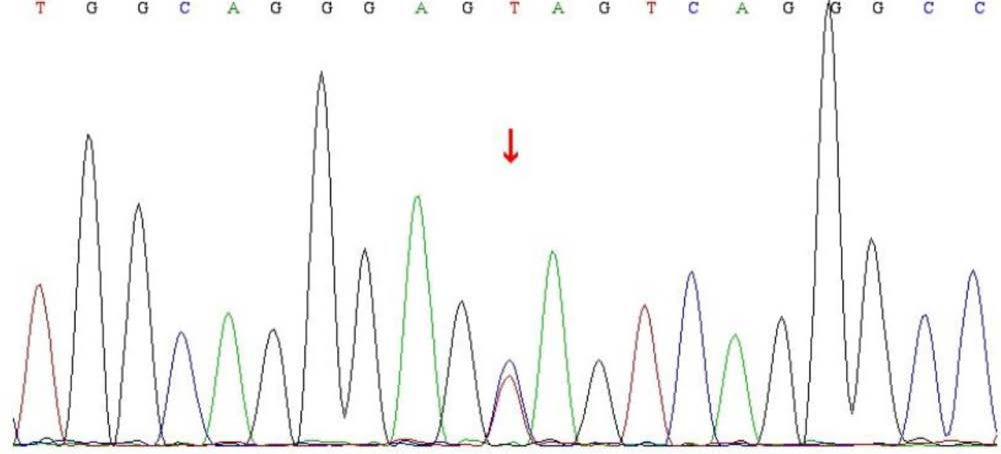
Genetic testing of the NOTCH3 gene reveals a point mutation at nucleotide 350, with a substitution of guanine (G) by adenine (A), denoted as c.350G > A.

A 34-year-old male first presented to the outpatient department of our hospital with a complaint of transient speech disorder accompanied by weakness in the left side of the body for 1 day in June 2020. The weakness in the patient left limbs lasted for 30 minutes before resolving spontaneously. Upon arrival at the hospital, only mild speech impairment persisted. The patient had no significant medical history or family history. The neurological examination revealed mild dysarthria, no evident abnormalities in cranial nerve examination, normal motor strength in all limbs, normal coordination, and normal deep and superficial sensations, with negative bilateral pathological signs. Post-admission, a cranial magnetic resonance imaging (MRI) was performed, and the diffusion weighted imaging (DWI) sequence showed a high-signal intensity in the right centrum semiovale, suggestive of acute ischemic infarction (Fig. 1A), along with multiple abnormal white matter hyperintensities in the brain. The T2-weighted and fluid-attenuated inversion recovery sequences demonstrated symmetrical diffuse white matter hyperintensities around the bilateral ventricles and deep white matter, as well as multiple lacunar infarcts affecting the external capsule, temporal pole, and frontal lobe (Fig. 1B–D). Cranial magnetic resonance angiograph showed no abnormalities in the intracranial arteries (Fig. 1E). Blood tests also revealed no obviously abnormalities. Given the clinical manifestationsn and MRI characteristics of the brain, CADASIL was considered. Genetic sequencing identified a heterozygous mutation in the NOTCH3 gene: a guanine to adenine substitution at nucleotide 350 (c.350G > A) (Fig. 2), which caused an amino acid change from cysteine to tyrosine at position 117 (p.C117Y). The patient was definitively diagnosed with CADASIL and received symptomatic treatment including antiplatelet aggregation therapy, lipid regulation, and plaque stabilization, resulting in improved symptoms.

In August 2021, the patient returned with recurrent episodes of visual blurring and abnormal limb sensation. A follow-up cranial MRI revealed an increase in symmetrical white matter signal abnormalities compared to 2020 (Fig. 1F–H), while no new-onset ischemic infarctions evident can be found on the DWI sequence.

In September 2023, the patient presented again with sudden cognitive dysfunction, mainly characterized by a decline in both short-term and long-term memory, computational impairment (unable to solve 1 + 1=?), and disorientation regarding people and places. A cranial MRI revealed a high signal in the right frontal lobe on the DWI sequence, suggesting cerebral acute ischemic infarction (Fig. 1I), with more extensive white matter signal abnormalities than before (Fig. 1J–L). During the hospital stay, treatment with antiplatelet therapy, lipid regulation, and acetylcholinesterase inhibitors led to a marked improvement in the patient memory, computational abilities, and orientation to people and places. We have summarized the characteristics of principal clinical symptoms, newly presented signs, mode of onset, imaging findings and cognitive function in Table 1.

**Table 1:** Summary of patient clinical data.

Date	Principal clinical symptoms	Newly presented signs	Mode of onset	Imaging findings	Cognitive function
2020.5	Intermittent episodes of slurred speech accompanied by left-sided limb weakness	Articulation disorder	Acute ischemic stroke	Right corpus callosum acute cerebral infarction lesion	MMSE 29 MoCA 22
2021.8	Recurrent episodes of blurred vision in conjunction with abnormal sensory perception in the left-sided extremities	Recent memory decline	Transient ischemic attack	Multiple abnormal signals in cerebral white matter, slightly increased compared to 2020	MMSE 27 MoCA 20
2023.9	Acute onset of cognitive impairment	Impaired proximal and distal memory, calculation disabilities (e.g., difficulties with simple sums such as 1 + 1=?), Challenges in person and place orientation, normal temporal orientation	Acute ischemic stroke with cognitive impairment	Acute infarction in the right frontal lobe accompanied by multifocal white matter hyperintensities, which have slightly increased since 2021.	Post-recovery: MMSE 24 MoCA 19

MMSE = mini-mental state examination, MoCA = montreal cognitive assessment.

## 3. Discussion

The origins of CADASIL can be traced back to 1977. It is a rare hereditary cerebral small vessel disease, attributed to mutations in the NOTCH3 gene located on chromosome 19. The NOTCH3 gene is instrumental in encoding cellular membrane receptors, crucially modulating vascular smooth muscle cells.^[3]^ Mutations within the NOTCH3 gene result in the aberrant formation of cysteine residues in epidermal growth factor-like repeat domains, thereby disrupting disulfide bond formation and altering protein conformation, which in turn precipitates the degeneration of smooth muscle cells via cytotoxic mechanisms. More than 280 distinct pathogenic mutations have been reported to date, predominantly missense mutations, inherited in an autosomal dominant fashion. Although the genetic defect associated with CADASIL was identified early on, the precise pathomechanisms remain somewhat elusive. Initial epidemiological investigations estimated the prevalence of CADASIL to be between 1.3 and 4.1 per 100,000 individuals, leading to its classification as an exceedingly rare condition.^[4]^ However, recent comprehensive genomic research has revealed a significantly higher prevalence of pathogenic NOTCH3 variants in the general population, with a striking incidence of 3.4 per 1000 individuals, particularly amongst Asian demographics.^[5]^ As a populous nation in Asia, it is incumbent upon neurologists and general physicians in China to acquire an in-depth comprehension of CADASIL clinical profile, biomarkers, imaging characteristics, and advancements in treatment strategies.

The onset and clinical manifestations of CADASIL vary greatly among patients. The most classic symptoms include: Migraine with typical aura; Subcortical ischemic stroke; Mood and psychiatric disorders; Cognitive deficits.^[6]^In addition to these classic symptoms, less common clinical presentations have been documented in the literature, such as gait abnormalities, urinary incontinence, epilepsy, cerebral hemorrhage, and pseudobulbar palsy.^[7]^ It is believed that racial differences and genetic variations may contribute to the variability in clinical symptoms observed in CADASIL patients. For instance, the proportion of CADASIL patients experiencing cerebral hemorrhage is only 0.5% to 2.4% in Europe. In contrast, the prevalence among CADASIL patients in Korea and Taiwan can reach 17% to 25% and 21%, respectively, with the majority of cases involving the p.Arg544Cys mutation.^[8,9]^ Interestingly, the incidence of migraines in the Asian CADASIL population is significantly lower than that in the European population.^[10]^The age range at which CADASIL manifests is broad, with some patients even presenting in old age, which is relatively uncommon for hereditary systemic diseases. Lee reported a case of an 86-year-old male who first showed symptoms of a cerebral infarction, later found to be associated with the p.Arg544Cys mutation.^[11]^ While several studies are underway to predict the clinical prognosis of CADASIL patients based on genetic mutations, it is currently unclear whether there is a significant genotype-phenotype correlation.

Neurofilaments play a pivotal role in the structural integrity of axons, consisting of chains of varying molecular weights, categorized as light, medium, and heavy. When axonal injury occurs, these components are released into the extracellular fluid, facilitating their quantification in cerebrospinal fluid or blood. In recent years, as research on biomarkers for CADASIL has advanced, neurofilament light chains have been recognized as a highly promising diagnostic marker. Numerous studies have established a substantial correlation between neurofilament light chain levels and the clinical symptoms of CADASIL, as well as serving as a prognostic indicator during both acute and chronic stages of ischemic stroke.^[12]^ Notably, neurofilament light chain concentrations are significantly elevated in CADASIL patients when compared to healthy individuals. However, the utility of neurofilament light chains as a solitary biomarker is often compromised by a lack of specificity, necessitating their combined use with other clinical parameters or biomarkers for a more comprehensive analysis.

Cranial MRI is instrumental in assessing the severity of CADASIL, differentiating it from other common ischemic cerebral strokes, and holds considerable value in prognosticating clinical outcomes. Imaging in CADASIL patients displays characteristic specificity, with MRI findings including subcortical infarcts, white matter hyperintensities, lacunar infarcts, microbleeds, and brain atrophy.^[13]^ In the early stages of the disease, white matter hyperintensities may initially appear around the ventricles or the centrum semiovale on T2-weighted or fluid-attenuated inversion recovery sequences, often leading to misdiagnosis as multiple sclerosis. As the disease progresses, the white matter hyperintensities gradually extend symmetrically, eventually involving the external capsule, temporal pole, and frontal lobe, areas more sensitive and specific to the disease. Given the diverse clinical manifestations of CADASIL and the general lack of comprehensive understanding of the disease among primary care physicians, misdiagnosis is common. Experienced specialists are required to integrate family history of migraines, ischemic strokes, and dementia, characteristic MRI findings, and genetic testing results to make a comprehensive assessment and definitive diagnosis.

Currently, the clinical treatment options for CADASIL are very limited and controversial. The European Academy of Neurology consensus on monogenic stroke treatment suggests not recommending thrombolytic therapy for lacunar strokes in CADASIL patients.^[14]^ However, the European Stroke Organization new guidelines for thrombolysis in acute ischemic stroke indicate that there is no strong evidence to avoid thrombolytic therapy in cases of mild stroke.^[15]^ Literature reports that CADASIL patients have undergone thrombolytic treatment post-acute ischemic stroke without a hemorrhagic transformation.^[16]^ The benefits of antiplatelet drugs in preventing ischemic strokes related to CADASIL are also not yet clear.^[17]^ Despite these controversies, most neurologists still treat CADASIL patients with antiplatelet drugs like aspirin or clopidogrel. Other measures applicable to the prevention of ischemic stroke may also apply to CADASIL patients, including quitting smoking and alcohol, controlling weight, managing blood lipids, regulating blood pressure, controlling blood sugar, and using statins. In terms of treatment for cognitive impairment, a multicenter randomized controlled trial involving 168 patients found that donepezil did not significantly improve symptoms of vascular dementia.^[18]^ Recent animal model studies indicate that administering hematopoietic growth factors, such as stem cell factor and granulocyte colony-stimulating factor, either alone or in combination, may have potential benefits during the acute, subacute, or even chronic stages of ischemic stroke.^[19,20]^

Our research also has certain limitations. This case demonstrates that CADASIL can recur multiple times in a patient with different clinical symptoms throughout the course of the disease, and some clinical symptoms may even be very atypical. However, it does not mean that all CADASIL patients have a similar course of disease. On the contrary, CADASIL with multiple different onset symptoms is very rare in clinical practice.

## 4. Conclusion

In summary, CADASIL can exhibit different clinical symptoms in different patients, as well as multiple different onset symptoms in 1 patient. Clinicians should consider the possibility of CADASIL in young patients with sudden typical neurological dysfunction including migraine with typical aura, subcortical ischemic stroke, mood and psychiatric disorders, and cognitive deficits.

## Acknowledgments

We would like to thank the patient and his family.

## Author contributions

**Conceptualization:** Siting Wu, Jiacai Lin.

**Data curation:** Ning Zhao, Xianli Sun, Jiacai Lin.

**Investigation:** Ning Zhao, Tingting Sun, Xianli Sun, Jiacai Lin.

**Project administration:** Jiacai Lin.

**Resources:** Tingting Sun, Fang Cui, Jiacai Lin.

**Supervision:** Tingting Sun.

**Validation:** Fang Cui.

**Visualization:** Jiacai Lin.

**Writing – original draft:** Siting Wu, Ning Zhao.

**Writing – review & editing:** Jiacai Lin.
